# 20-Year Efficacy of Endoscopic Thoracic Sympathectomy for Primary Hyperhidrosis: A Cohort Study

**DOI:** 10.3390/jcm14144831

**Published:** 2025-07-08

**Authors:** Anna Ureña, Leandro Grando, Lluisa Rodriguez-Gussinyer, Ivan Macía, Francisco Rivas, Nestor Iván Quiroga, Camilo Moreno, Xavier Michavilla, Ricard Ramos

**Affiliations:** 1 Department of Thoracic Surgery, Hospital Clinic of Barcelona, Universitat de Barcelona, 08036 Barcelona, Spain; aurenal@clinic.cat (A.U.); leandroezequiel.grando@hmar.cat (L.G.); quiroga@clinic.cat (N.I.Q.); xollerm@clinic.cat (X.M.); 2 Institut d’Investigacions Biomèdiques August Pi i Sunyer-IDIBAPS, 08036 Barcelona, Spain; 3 Facultat de Medicina, Universitat de Barcelona, 08907 Barcelona, Spain; lluisarg@gmail.com; 4 Department of Thoracic Surgery, Hospital Universitari de Bellvitge, Universitat de Barcelona, 08907 Barcelona, Spain; imacia@bellvitgehospital.cat (I.M.); frivas@bellvitgehospital.cat (F.R.); cmorenom@bellvitgehospital.cat (C.M.)

**Keywords:** hyperhidrosis, compensatory sweating, long-term satisfaction, anxiety

## Abstract

**Background:** Primary focal hyperhidrosis, characterized by excessive sweating primarily affecting the hands and axillae, can significantly impact quality of life. Bilateral thoracic sympathectomy is the gold standard treatment, providing permanent resolution of palmar hyperhidrosis. Most studies evaluating outcomes and patient satisfaction after sympathectomy focus on short- to medium-term follow-up, typically up to 5 years. This study aimed to assess anxiety, satisfaction, and sweat redistribution 20 years after bilateral endoscopic thoracic sympathectomy. **Methods**: Between January 2002 and December 2003, 106 patients with primary hyperhidrosis underwent bilateral endoscopic thoracic sympathectomy targeting ganglia T2–T3 at our center. The patients were contacted via telephone in 2023 and asked to complete the same survey they had filled out preoperatively and 12 months postoperatively. The survey evaluated sweat redistribution, anxiety, hyperhidrosis-related symptoms, and satisfaction. Anxiety, satisfaction, and perceived sweating were rated using a 5-point visual analog scale (VAS) ranging from 0 (unsatisfied/no symptoms) to 4 (very satisfied/severe symptoms). **Results**: Of the 106 eligible patients, 24 (22.6%) completed the survey. Most reported persistent anhidrosis (palmar or palmar–axillary) 20 years post-surgery. The survey results remained consistent with those at the 1-year follow-up. Significant sweat redistribution to the abdomen and back was observed. Patient satisfaction remained high, with no significant differences between the 1-year and 20-year assessments. Anxiety significantly decreased compared to preoperative levels (*p* < 0.001). **Conclusions**: Bilateral endoscopic thoracic sympathectomy is an effective long-term treatment for reducing excessive sweating. Patient satisfaction remains high over time, despite the persistence of compensatory sweating.

## 1. Introduction

Primary hyperhidrosis (PH) is a chronic condition characterized by excessive, focal sweating beyond physiological needs, most commonly affecting the palms, axillae, soles, or craniofacial region [1]. The global prevalence is estimated at 2.8–5%, with a notable psychosocial burden, especially in young adults [2]. Although the underlying mechanisms remain incompletely understood, PH is generally attributed to hyperactivity of the sympathetic nervous system, particularly in the upper thoracic ganglia [3].

PH significantly impairs patients’ quality of life (QoL), not only through physical symptoms but also due to its psychological and social impacts. Affected individuals often report elevated anxiety, social withdrawal, and diminished self-esteem, which may hinder academic, professional, and interpersonal development [4]. Various non-surgical treatment options exist, including topical aluminum chloride, iontophoresis, botulinum toxin injections, and systemic anticholinergics, but these typically offer only temporary relief or are limited by tolerability and adherence issues [5].

For patients with severe or refractory symptoms, endoscopic thoracic sympathectomy (ETS) is considered the most effective long-term solution. ETS involves interruption of the thoracic sympathetic chain at specific levels (commonly T2–T3 for palmar hyperhidrosis), resulting in durable anhidrosis in the target region [6,7]. However, the development of compensatory sweating (CS)—increased perspiration in untreated areas such as the abdomen or back—is a frequent and often unavoidable side effect, with variable influence on overall satisfaction [8,9,10,11].

While multiple studies have demonstrated the safety and efficacy of ETS in the short- to medium-term (up to 5 years postoperatively), long-term data remain limited [12,13,14]. Key questions persist regarding the durability of anhidrosis, evolution of CS, and the maintenance of psychological benefits such as anxiety reduction. Additionally, there is a paucity of longitudinal research evaluating these outcomes within the same patient cohort across extended time frames.

The present study addresses this gap by examining a cohort of patients who underwent ETS for PH at our institution between 2002 and 2003. Using a validated and consistently applied questionnaire, we assessed clinical outcomes, sweat distribution, anxiety levels, and satisfaction before surgery, at 1 year, and again approximately 20 years after intervention. This extended follow-up provides a unique opportunity to evaluate the long-term stability of both the therapeutic benefits and side effects of ETS in PH.

## 2. Materials and Methods

### 2.1. Patients

A retrospective review was conducted using a prospectively maintained institutional database to identify patients who underwent endoscopic bilateral thoracic sympathectomy for primary palmar, axillary, or palmoaxillary hyperhidrosis between January 2002 and December 2003. Inclusion criteria were age ≥ 16 years at the time of surgery, diagnosis of primary hyperhidrosis, and availability of complete preoperative and one-year follow-up data. Exclusion criteria included patients with secondary hyperhidrosis, a history of prior thoracic procedures, or known psychiatric disorders not related to hyperhidrosis.

### 2.2. Methods

Questionnaire Survey

To assess the clinical impact of surgery, a structured, hospital-validated questionnaire specifically designed for primary hyperhidrosis was used. This instrument was previously developed and applied in earlier prospective studies by our group [6] and evaluates sweating distribution, symptom severity, associated psychosomatic symptoms, perceived anxiety, and satisfaction with the surgical outcome.

**Figure 1:**
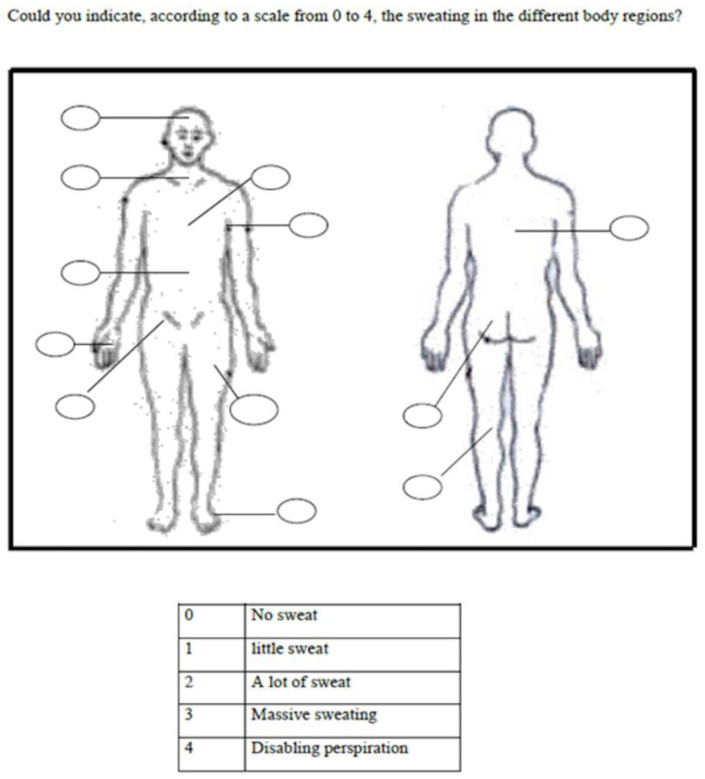
Diagram of the human body used for the patients to indicate the amount of sweating in the different regions on a 5-point scale (VAS) ranging from 0 (no sweating) to 4 (disabling sweating).

During the initial preoperative assessment phase (2002–2003), all patients completed the questionnaire approximately eight weeks prior to surgery. A visual diagram of the human body was included (Figure 1), allowing the patients to indicate the intensity of sweating in predefined regions: hands, axillae, feet, back, and abdomen. Severity was rated using a five-point visual analog scale (VAS), ranging from 0 (no sweating) to 4 (disabling hyperhidrosis). Regions scored as 0 were considered anhidrotic.

**Figure 2:**
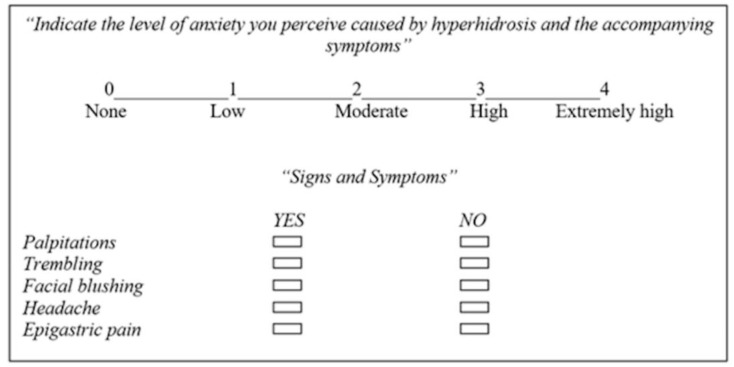
Scale used to assess level of anxiety.

Anxiety was assessed using a separate VAS ranging from 0 (no anxiety) to 4 (extremely high anxiety). Additionally, the presence of symptoms commonly linked to hyperhidrosis—such as palpitations, hand tremors, facial blushing, headache, and nonspecific epigastric discomfort—was documented (Figure 2). All responses were anonymized and securely stored for future analysis.

A structured postoperative assessment using the same questionnaire was carried out at one year. This follow-up excluded questions related exclusively to the preoperative period but retained the core sections on sweat distribution, anxiety, related symptoms, and satisfaction with treatment. Satisfaction was graded on a five-point VAS, from 0 (not satisfied) to 4 (very satisfied).

In 2023, a long-term follow-up was conducted through structured and scheduled telephone interviews. The same original questionnaire was used to maintain consistency across all time points. Trained staff, blind to previous scores, performed the interviews. The goal was to reassess the distribution of sweating, current anxiety levels, and long-term satisfaction, enabling direct comparison with the preoperative and one-year data and, thus, evaluating the persistence of treatment effects over two decades.

### 2.3. Surgical Technique

All surgical procedures were performed under general anesthesia with selective (double-lumen) intubation to allow for single-lung ventilation. The patients were positioned in a semi-sitting (25°) supine position with the upper limbs abducted to facilitate thoracoscopic access. A single 10 mm trocar was inserted in the third intercostal space at the mid-axillary line to introduce the thoracoscope and instrumentation.

The sympathetic chain was identified, and electrocoagulation of the T2 and T3 ganglia was performed using monopolar forceps at a power setting of 25 W. The chain was transected over the ribs to ensure complete interruption of sympathetic outflow. A routine chest X-ray was obtained postoperatively to exclude pneumothorax. If no complications were observed, the patients were discharged within 17–24 h post-surgery.

## 3. Results

### 3.1. Patient Characteristics

A total of 112 patients underwent endoscopic bilateral thoracic sympathectomy for primary palmar, axillary, or palmoaxillary hyperhidrosis during 2002 and 2003. In total, 106 patients (76 women [72%] and 30 men [28%]; mean age 28.4 years, range 17–55 years) completed both the preoperative and one-year postoperative assessments.

Two (1.9%) patients died due to cardiovascular disease and a traffic accident. Fifty-seven (53.8%) patients, according to the hospital database, changed addresses, resulting in the loss of contact information. In twenty-three (21.7%) patients, we only had a mailing address and a non-mobile phone number, and we were unable to contact them.

The anonymized database contained only telephone numbers recorded at the time of surgery, approximately 20 years earlier, and attempts to recover updated phone numbers from medical records were unsuccessful. No selection bias occurred because all surviving patients were contacted to the extent possible. A postal mail campaign was also considered but was not feasible due to address changes over the two-decade period.

We successfully contacted 24 of the 106 patients (22.6%); of these, 17 were women (70.8%), and 7 (29.2%) were men. The time that passed between the surgery and the survey ranged from 20 to 22 years. All contacted patients agreed to participate in the structured telephone interview.

### 3.2. Localization and Redistribution of Hyperhidrosis

**Figure 3:**
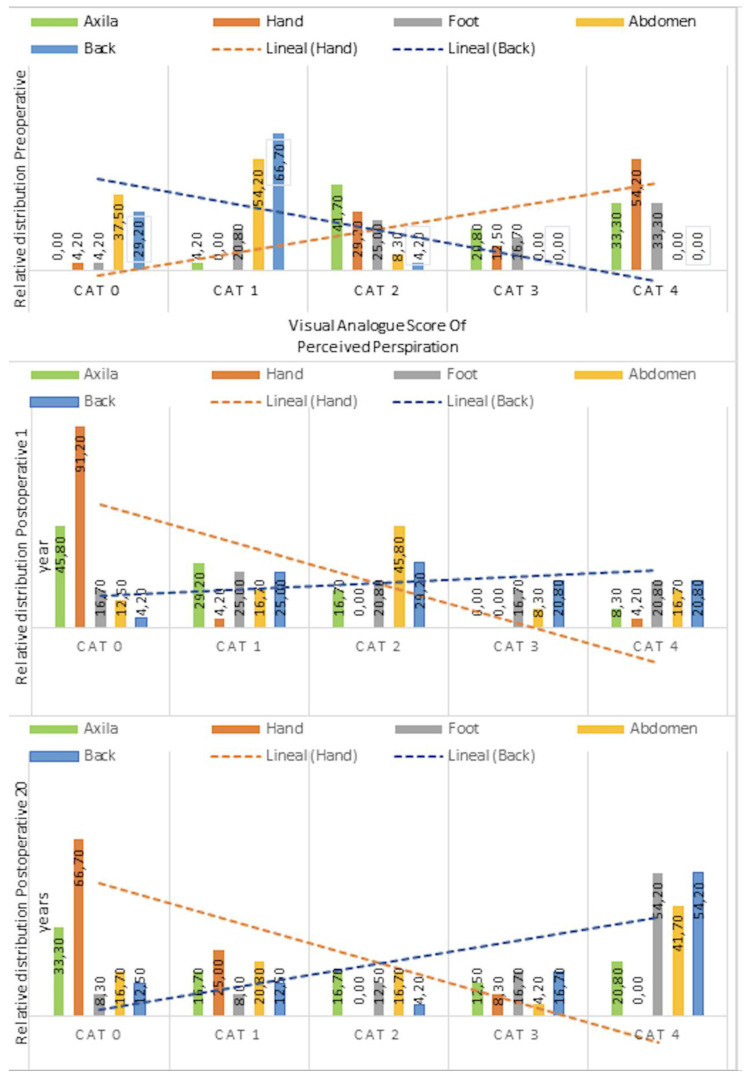
Anatomical distribution of sweating and perceived degree of perspiration before and after surgery. Patients rated their perceived perspiration on a 5-point visual analogue scale ranging from 0 (no perspiration) to 5 (excessive perspiration).

The new survey revealed a redistribution of sweating at 20 years (Table 1 and Figure 3). A complete reduction was observed in sweating on the palm of the hand (the main reason for performing the surgery) immediately after the surgery, which persists after 20 years. Additionally, a reduction in underarm sweating was also observed. On the other hand, an increase in sweating in areas such as the abdomen was noted.

### 3.3. Level of Anxiety and Satisfaction

Table 2 shows the changes in hyperhidrosis-related anxiety levels at all three time points. The level of anxiety decreased significantly over time from a mean (± SD) of 2.08 ± 1.1 at the preoperative assessment to 0.39 ± 0.67 (*p* < 0.001) at 1 year and 0.16 ± 0.81 at 20 years (*p* < 0.001). The mean (SD) degree of satisfaction at one year was 3.33 ± 0.70. Satisfaction remained high at the 20-year evaluation (3.04 ± 1.30).

### 3.4. Associated Symptoms with Hyperhidrosis

We compared the number (%) of patients reporting the following symptoms at baseline versus one-year after surgery, and all of the comparisons were statistically significant: palpitations: 11 (45.8%) vs. 7 (29.1%), *p* < 0.05; trembling of the hands: 6 (25%) vs. 1 (4.2%), *p* < 0.05; facial blushing: 15 (62.5%) vs. 7 (29.1%), *p* < 0.05; headache 8 (33.3%) vs. 2 (8.3%), *p* < 0.001; and nonspecific epigastric pain: 5 (20.8%) vs. 1 (4.2%), *p* < 0.05.

At 20 years, most of these symptoms had been resolved. Only two patients (8.3%) reported palpitations and facial blushing, and one patient (4.2%) reported hand tremors and headache. None of the patients reported nonspecific epigastric pain (*p* < 0.001).

## 4. Discussion

The present study shows that sympathectomy is an effective long-term treatment for primary palmar and palmar–axillary hyperhidrosis, even 20 years after surgery. However, palmar anhidrosis was frequently associated with the redistribution of sweating in the immediate postoperative period and in the medium to long term. The degree and distribution of compensatory sweating observed in this study is consistent with previous reports [12].

These results are consistent with those reported by Shabat et al. [13], who described excellent long-term patient satisfaction despite the frequent occurrence of compensatory sweating (CS) in a cohort followed for more than 15 years. Similarly, Askari et al. [14] reported sustained efficacy and satisfaction at 16 years post-surgery in a UK-based population. The magnitude and distribution of compensatory sweating observed in our series—most notably affecting the abdomen and back—are comparable to these prior reports, supporting the notion that CS represents a persistent but generally tolerated side effect of ETS in the long term.

Anxiety is a common symptom in patients with primary hyperhidrosis. Most patients have a high level of anxiety before surgery. However, anxiety decreases significantly after the surgery, despite the emergence of compensatory sweating [7,9,12,14]. The body of evidence to support thoracic sympathectomy for the treatment of hyperhidrosis continues to grow, including a recent study by Lima et al. [15], who reported a prospective study evaluating the quality of life (QoL) in young patients with primary hyperhidrosis (PH) before and after endoscopic thoracic sympathectomy. The authors reported significant improvements in QoL scores postoperatively, with marked reductions in both physical symptoms and psychosocial distress. Notably, patient satisfaction remained high despite the occurrence of compensatory sweating in a substantial proportion of cases.

In the present study, which continues a line of research begun in 1998 with an encrypted database, we evaluated the same patient cohort that we had initially treated 20 years ago. We used the original questionnaire to assess changes over this 20-year period, which was administered at three time points—baseline (preoperative), 1-year post-sympathectomy, and at 20 years. Specifically, we evaluated changes in sweating according to the anatomical region, degree of anxiety and its repercussions, and associated symptoms. Our findings confirm previous reports [16,17,18] that reported substantial improvements in quality of life (QoL) and reductions in hyperhidrosis-related anxiety after surgery but, importantly, with a much longer follow-up period.

Kobayashi et al. (2024) [16] and Turhan et al. (2022) [17] both reported their long-term experiences with thoracoscopic sympathectomy and sympathicotomy, respectively, focusing on patient-reported outcomes over a mean follow-up period of 10 years. Both studies found that palmar anhidrosis was sustained in the majority of patients, with high levels of overall satisfaction maintained throughout the follow-up period. Despite the frequent occurrence of compensatory sweating—most notably affecting the trunk—patients generally adapted well over time. In the series by Turhan et al., the intensity of compensatory sweating tended to decrease in many patients as the years passed, contributing to stable satisfaction rates. These findings reinforce the long-lasting efficacy of the procedures and underscore the need for thorough preoperative counseling to set realistic expectations regarding both the benefits and potential long-term side effects. In line with these previous reports, we found that anhidrosis in the hands and axillae was maintained over time, but there was a compensatory increase in sweating in the back, abdominal region, and feet.

The reduction in anxiety observed at 1-year remained unchanged after 20 years and significantly below preoperative levels, despite the presence of compensatory sweating. Importantly, compensatory sweating did not affect overall satisfaction, which remained high and virtually unchanged from the one-year assessment. This finding confirms the effectiveness of T2-T3 thoracic sympathectomy at 1 and 20 years after surgery. Palmar and axillary anhidrosis were maintained over time, with high patient satisfaction [19].

Numerous studies have attempted to determine the association between compensatory sweating and the level at which the thoracic sympathetic chain is cut (T2, T3, T4, or a combination of these) [20,21,22]. A meta-analysis of 11 randomized controlled trials (1079 patients) concluded that restricting or lowering the level of sympathectomy (e.g., preserving T2 or targeting lower levels) does not significantly reduce the incidence or severity of CS [20]. In this regard, the optimal point for resection of the sympathetic chain is controversial. However, in this study, our aim was to evaluate patients who underwent surgery in the early 2000s, when the recommendation was to cut the sympathetic chain at the T2-T3 level.

The main limitation of this study is related to the telephone recruitment of patients. Given that 20 years had passed since the surgical intervention, we were unable to contact all the participants, probably because the telephone number (typically a landline) registered 20 years ago was no longer in use, either because the patients had moved or switched to a mobile telephone with a new number. By contrast, an important strength of this study was that the same questionnaire was administered at all three time points, which allowed us to directly compare all the study variables. Another strength is that the long period of time between administration of the questionnaire meant that the patients were unlikely to have remembered the questions and, thus, responded as if it were a new questionnaire.

In conclusion, this study confirms that endoscopic thoracic sympathectomy offers a long-term solution to hyperhidrosis, despite the presence of compensatory sweating. Moreover, the reduced anxiety and high satisfaction associated with treatment are maintained over time.

## Figures and Tables

**Table 1:** Anatomical distribution and perceived degree of hyperhidrosis before and after surgery.

Anatomical Region	Preoperative (n = 24)	1 Year Postoperative (n = 24)	20 Years Postoperative (n = 24)	*p* Value
	Median (IQR)			
**Axilla**	3.0 (2.0, 4.0)	1.0 (0.0, 1.2)	1.5 (0.0, 3.0)	<0.001
**Hand**	4.0 (2.0, 4.0)	0.0 (0.0, 0.0)	0.0 (0.0, 1.0)	<0.001
**Foot**	2.5 (1.8, 4.0)	2.0 (1.0, 3.0)	4.0 (2.0, 4.0)	0.053
**Abdomen**	1.0 (0.0, 1.0)	2.0(1.0, 2.2)	2.0 (1.0, 4.0)	<0.001
**Back**	1.0 (0.0, 1.0)	2.0 (1.0, 3.0)	4.0 (1.8, 4.0)	<0.001

Patients rated their perceived perspiration on a 5-point visual analogue scale ranging from 0 (no perspiration) to 5 (excessive perspiration).

**Table 2:** Changes in anxiety levels and degree of satisfaction from baseline (preoperative) to 1 and 20 years after endoscopic thoracic sympathectomy.

	Preoperative (n = 24)	1 Year Postop (n = 24)	20 Years Postop (n = 24)	*p* Value
	Mean ± SD			
**Anxiety level**	2 ±1.02	0.37 ± 0.57	0.16 ± 0.81	<0.001
**Degree of satisfaction**	NA	3.33 ± 0.70	3.04 ± 1.30	0.306

## Data Availability

The datasets generated during and/or analyzed during the current study are available from the corresponding author on reasonable request.

## Abbreviations

The following abbreviations are used in this manuscript:

PREOP	Preoperative
POSTOP	Postoperative
PH	Primary hyperhidrosis
ETS	Endoscopic thoracic sympathectomy
QoL	Quality of life
IQR	Interquartile range
SD	Standard deviation
NA	Not applicable
VAS	Visual analogue scale
CS	Compensatory sweating
